# Evidence Acquisition and Evaluation for Evidence Summit on Enhancing Provision and Use of Maternal Health Services through Financial Incentives

**Published:** 2013-12

**Authors:** Elizabeth S. Higgs, Emily Stammer, Rebecca Roth, Robert L. Balster

**Affiliations:** ^1^National Institute of Allergy and Infectious Diseases, Bethesda, MD, USA; ^2^United States Agency for International Development, Washington, DC, USA; ^3^Knowledge Management Services, Washington, DC, USA; ^4^Virginia Commonwealth University, Richmond, VA, USA

**Keywords:** Incentive, Maternal health services, Maternal mortality, Motivation, Perinatal mortality, Prenatal care, Reimbursement

## Abstract

Recognizing the need for evidence to inform US Government and governments of the low- and middle-income countries on efficient, effective maternal health policies, strategies, and programmes, the US Government convened the Evidence Summit on Enhancing Provision and Use of Maternal Health Services through Financial Incentives in April 2012 in Washington, DC, USA. This paper summarizes the background and methods for the acquisition and evaluation of the evidence used for achieving the goals of the Summit. The goal of the Summit was to obtain multidisciplinary expert review of literature to inform both US Government and governments of the low- and middle-income countries on evidence-informed practice, policies, and strategies for financial incentives. Several steps were undertaken to define the tasks for the Summit and identify the appropriate evidence for review. The process began by identifying focal questions intended to inform governments of the low-and middle-income countries and the US Government about the efficacy of supply- and demand-side financial incentives for enhanced provision and use of quality maternal health services. Experts were selected representing the research and programme communities, academia, relevant non-governmental organizations, and government agencies and were assembled into Evidence Review Teams. This was followed by a systematic process to gather relevant peer-reviewed literature that would inform the focal questions. Members of the Evidence Review Teams were invited to add relevant papers not identified in the initial literature review to complete the bibliography. The Evidence Review Teams were asked to comply with a specific evaluation framework for recommendations on practice and policy based on both expert opinion and the quality of the data. Details of the search processes and methods used for screening and quality reviews are described.

## INTRODUCTION

Over their lifetime, women are about 100 times more likely to die as a result of pregnancy in sub-Saharan Africa than in developed regions of the world (1). These enormous discrepancies highlight vast inequity as well as the possibility of lowering preventable maternal mortality to a great extent. Yet, women in low- and middle-income countries (LMICs) with high burden of maternal death often underutilize the maternal health services intended to facilitate healthy births and protect maternal lives. Creating demand for lifesaving services is a challenge shared across many areas of global health but it is particularly compelling in maternal health, given the high mortality rates among mothers and the children they leave behind. Financial barriers contribute to both underutilization and lack of availability of potentially lifesaving services.

A renewed emphasis on the application of research and evaluation to inform strategic thinking about development for LMICs is integral to the US Government's efforts to improve health by promoting country-owned, effective and sustainable interventions. To that end the US Agency for International Development (USAID) is leading a series of evidence summits focused on important development challenges. The aim of these summits is to provide evidence-based expert recommendations on how to achieve some of the world's most difficult development goals, for example, caring for children living outside families (2) and supporting community health workers (3).

To address the problem of financial barriers to maternal healthcare utilization, the US Government convened the Evidence Summit on Enhancing Provision and Use of Maternal Health Services through Financial Incentives on 24-25 April 2012 in Washington, DC. The Summit brought together leading researchers, development experts, and those involved in implementing programmes in the field to assess the evidence that will ultimately inform policies, strategies, and programmes relevant to financial incentives and maternal health services in LMICs and, in so doing, simultaneously identify gaps in evidence that could shape the future research agenda. This Evidence Summit reflects USAID's commitment to evidence-based, innovative, efficacious, effective and sustainable development efforts of the US Government in partnership with other governments. The rapid application of knowledge and scale-up of novel discoveries and innovations to populations needing them most requires a continuum of learning from basic to operational research and a broad coalition of expertise and contributors across the US Government, academia, and policy- and practice-related leaderships from developing countries. The Eunice Kennedy Shriver National Institute of Child Health and Human Development at the National Institutes of Health joined USAID in the organization and support of this Evidence Summit. This paper describes the Evidence Summit process from its inception to the post-summit activities. Other papers in this Supplement of the Journal present the findings and recommendations.

**Figure 1.**
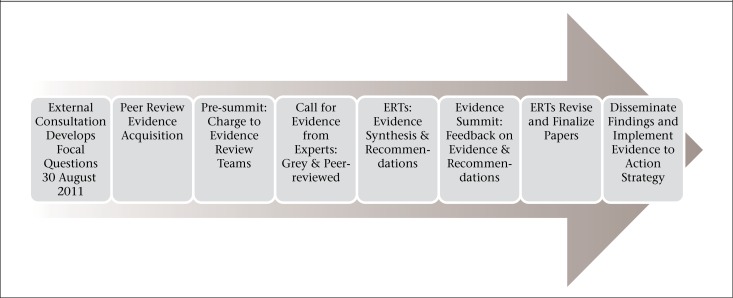
Diagram of the Evidence Summit process from initial organization to activities, still ongoing, to implement the recommendations

### Overview of process of the Evidence Summit

The initial planning for the Summit originated in the Bureau for Global Health at USAID and was tied to President Obama's Global Health Initiative (http://www.ghi.gov/), which calls for interagency collaboration, innovation, and doing more of what works as guiding principles for US Government's assistance in global health. Like other evidence summits of the US Government, this Evidence Summit was not a single event, but rather a year-long process that led up to the April 2012 Summit and continues with the implementation of recommendations developed during the Summit process (Figure 1). Following the identification of the general topic of creating demand for maternal health services as a potential topic for the Evidence Summit, a Core Group of persons responsible for the overall organization and direction of the Summit was assembled with 19 experts in maternal health, economics, global health, research, and implementation drawn from three US Government agencies with ongoing activities in support of maternal health (http://www.usaid.gov/sites/default/files/documents/1864/mh_summit_interagency_planning_group.pdf). The formation of the Core Group was followed by a scoping exercise consisting of an external consultation, which included experts in maternal health, economics, and implementation to review the topic and identify key questions. Leveraging this external advice, the Core Group created focal questions to guide the evidence review process, the selection and appointment of external and internal experts to Evidence Review Teams (ERTs), a systematic literature search for documents relevant to the focal questions, and the screening and evaluation of these documents and the identification of additional relevant material by members of the ERTs. A pre-summit meeting was held to organize and motivate the ERTs, which subsequently prepared Evidence Synthesis Papers and drafted recommendations for presentation at the Summit. The final step of the Evidence Summit process—the revision of materials based on feedback at the Summit and the implementation of an Evidence to Action Plan to act upon recommendations—is still underway. The following sections provide more details on these steps in the Evidence Summit process, focusing primarily on the gathering of evidence and review phases.

## GETTING STARTED

### Selecting the topic and developing focal questions

The USAID's Bureau for Global Health identified topics for Global Health Evidence Summits, using a set of criteria with seven elements which reflected their commitment to evidence-informed global health decision-making and to engaging external expertise from multidisciplinary experts for complex development challenges:

1. Enough evidence on the topic is available to permit policy and/or programmatic decision-making;
2. Rigorous studies or systematic analyses are adequately represented in the body of available evidence on the topic;
3. The application of evidence will likely result in high impact (health outcomes) and/or improved implementation of interventions;
4. There is a strong demand from the field for evidence-based solutions to pressing policy/programme challenges or questions represented by the topic;
5. The topic informs an important global health issue;
6. The evidence on the topic can be pooled, synthesized, shared, and discussed at reasonable cost; and
7. Evidence-based guidance is not available on the topic or needs to be updated.

The underutilization of maternal health services was identified as a general topic for an evidence summit. Additional information on the scoping exercise and rationale for the topic can be found in the introductory paper to this Supplement (4). Recognizing the need to refine and narrow this topic further, the Core Group organized a scoping exercise titled “Barriers to the Use of Maternal Care: Antenatal Care, Skilled Birth Attendance, Facility Delivery, and Emergency Obstetric and Newborn Care.” Targeted questions on barriers to the utilization of maternal health services were posed to key informants from the Maternal Health Task Force, World Health Organization, Save the Children, UK Department for International Development, University of Aberdeen, World Bank, Family Care International, and the Bill & Melinda Gates Foundation. The scoping exercise identified financial barriers as key obstacles to maternal healthcare utilization. Based on these results, the Core Group refined the topic to “Utilizing Financial Incentives to Create Demand for Maternal Health Services.”

The Core Group met over a period of several months to draft a concept paper describing the background for the Summit, the goals and anticipated outcomes, and a process for accomplishing the summit objectives. On 30 August 2011, the Core Group also convened an external consultation with experts from the fields of maternal health and performance-based financing to review the concept paper and identify key questions for the Evidence Summit. Participants in the consultation included experts from the Population Council, Agency for Health Research and Quality, Broad Branch Associates, Center for Global Development, Abt Associates/Health Systems 20/20, and the Bill & Melinda Gates Institute for Population and Reproductive Health/Johns Hopkins Bloomberg School of Public Health. During the external consultation, the experts advised that supply- and demand-side interventions and outcomes were interdependent and should not be separated in the Evidence Summit. This advice resulted in additional refinements of the concept paper, including a decision to focus on financial incentives that would increase both demand for and supply of maternal healthcare services.

Following extended internal discussions and consideration of recommendations provided during the external consultation, the Core Group selected two key focal questions to be addressed during the summit process. These focal questions emerged from a set of beliefs about the roles of financial incentives, the contexts that led to those beliefs, and the formation of explicit development hypotheses about what the evidence review might find. These are as follows:

- *Focal Question 1:* What financial incentives, if any, are linked positively or negatively to maternal and neonatal health outcomes, the provision or utilization of maternal health services, or to care-seeking behaviour by women?
  - *Belief:* Financial incentives can influence users’ and providers’ behaviours, including the utilization and provision of services and can potentially alter maternal and neonatal health outcomes positively and, in some cases, negatively. Some incentives will be more influential than others and interaction of incentives in various combinations will produce different results.
  - *Context:* In recent years, financial incentives in the form of vouchers, waivers, conditional cash transfers, variations of pay-for-performance, and so forth, have galvanized tremendous interest in the public health community. Considerable documentation of financial incentives for health, in general, has been compiled. To date, there is less information relating to the effect of financial incentives on maternal health behaviours, including the use of services, providers’ behaviours, and maternal and neonatal health outcomes. Many governments and donors are supporting, with substantial investments, implementation of financial incentives for maternal and newborn health but this is based on limited evidence. Because of the significant potential to affect the use and provision of services, there is a need to identify, synthesize, and analyze the available evidence to determine positive and negative effects for maternal and newborn health.
  - *Development hypothesis:* A review of evidence of financial incentives and their effects on maternal and neonatal health behaviours, service delivery, and outcomes will increase understanding of available interventions and lead to more effective and efficient policies, programmes, and strategies.
- *Focal Question 2:* What are the contextual factors that impact the effectiveness of the financial incentives?
  - *Belief:* Numerous contextual factors, including household income and wealth, providers’ compensation, geography, health workers’ quality and access, availability of transport, capacity of services to accommodate more clients effectively, management of the financial incentive programme, quality of the health management information system, the political situation, and so forth, are critical to implementation of the incentive programmes for maternal health and for their results.
  - *Context:* While there is potential for significant positive changes for health behaviours and health outcomes, those with experience in implementing and evaluating financial incentive programmes to date advise that, without understanding the context in which the financial incentives are applied, it is difficult to generalize from results in any one setting. For example, a programme that quickly increases service utilization but that cannot provide quality services could result in fewer clients accessing services over time and/or yield negative health outcomes. Furthermore, supervision and support, social norms, household support (from husband/partner/mother/mother-in-law), community wealth (or wealth inequality), and infrastructure are some of the other contextual issues that can influence the results of financial incentive programmes.
  - *Development hypothesis:* A review of various levels of evidence about the wide range of contextual variants in financial incentive programmes will aid in understanding the nuances of designing and implementing policies and programmes for effective results in different settings.

**Figure 2.**
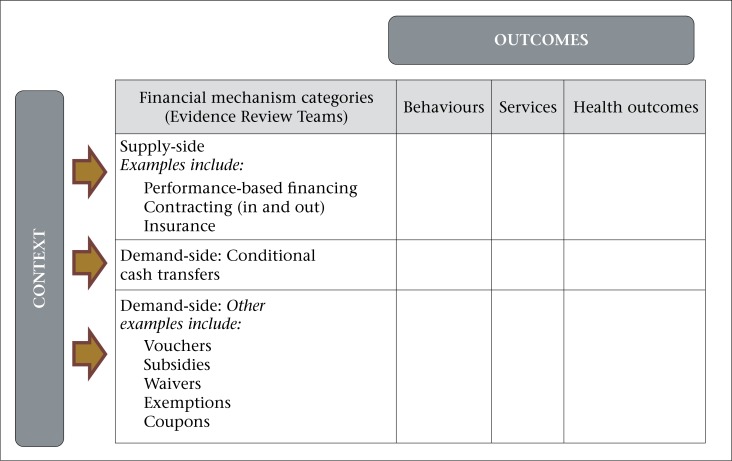
Diagram showing how the evidence was categorized for assignment to Evidence Review Teams

To facilitate the organization of the Evidence Summit and the literature search process, the Core Group subdivided the evidence by the type of financial incentive and development measure assessed in the study. As shown in Figure 2, the three groups of financial mechanisms were supply-side mechanisms, conditional cash transfers, and other demand-side mechanisms; the outcomes were maternal healthcare utilization behaviour, changes in the frequency, nature or quality of services provided, or the more distal outcomes of maternal and infant mortality and morbidity. The supply-side mechanisms, offered by governments, health systems, facilities, and NGOs include various forms of performance-based or outcome-based incentives to providers, direct fiscal transfers or financial supplements for service provision, contracting for services within or outside provider groups. Cash transfers to mothers or families conditional on increased utilization of health services are part of a global interest in conditional cash transfers in the area of social protection. The application to health services utilization has not been well-studied but represents a unique literature to warrant consideration in its own right. Many of the other demand-side incentives derive, in part, from the same notion that underlies conditional cash transfers. Those providing subsidies or vouchers exchangeable for goods and services or offsetting transportation or childcare costs if patients attend clinics form another type of financial incentive. Other examples are exemptions from payment or coupons to defray costs.

Finally, the careful examination of the context in which the research was conducted was critical to answering Focal Question 2. There are several dimensions along which context might be considered. Geographical region, nation, urban versus rural, or culture may be an important contextual factor influencing the outcomes of different financial incentives. It may be that certain characteristics of the patient, such as socioeconomic status or membership in a stigmatized group, could modify the effectiveness of incentives. The ERTs were asked to identify the contextual elements that should be examined to answer Focal Question 2.

### Formation of Evidence Review Teams (ERTs)

Central to the Evidence Summit process was the selection and organization of experts into three ERTs to assess the evidence on financial incentives and maternal health services and make recommendations on policies, strategies, and programmes. Experts were nominated by Core Group members or, in some cases, by outside experts who were initially contacted about participating but who nominated someone else in their place. Many members of the Core Group also served on ERTs. The challenge in selecting ERT members was to achieve balance along several dimensions, such as area and level of expertise, programmatic and research experience, and affiliation. ERT members had expertise in maternal health, health economics, health systems, development, and other related topics, and many had experience in implementing programmes or conducting research involving the use of financial incentives. They represented a mix of senior and mid-level managers, practitioners, and researchers. ERT members were affiliated with the following institutions:

- *Non-governmental organizations*: Abt Associates; Broad Branch Associates; Center for Global Development; Futures Institute; JHPIEGO; John Snow, Inc.; Population Council; Results for Development; RTI International; Save the Children; and University Research Co.
- *Academic institutions*: Johns Hopkins Bloomberg School of Public Health, Harvard School of Public Health, London School of Hygiene & Tropical Medicine, University of North Carolina, Gillings School of Global Public Health, and Virginia Commonwealth University
- *US Government agencies*: Centers for Disease Control and Prevention, National Institutes of Health, and USAID
- *Private foundation*: Bill & Melinda Gates Foundation
- *Bilateral and multilateral institutions*: Department for International Development (United Kingdom), World Bank, and World Health Organization.

Although the number of ERT members based in LMICs was relatively small, many experts had extensive experience working and residing in these settings. It was decided to bring a greater LMIC perspective into the process during the Summit itself by inviting participants from some of these countries. Finally, the Core Group selected ERT members based on their knowledge and expertise in maternal health services, financial incentives, health systems, health economics, development, and other related topics. A large majority of persons invited to become ERT members agreed to serve in this capacity, which was a significant effort commitment, given the long-term nature of the Evidence Summit process and the work required for these teams.

The three ERTs were organized around three categories of financial incentives: supply-side financial incentives, conditional cash transfers, and other demand-side financial incentives (excluding cash transfers) (Figure 2). The ERT members with specific expertise in a category of financial incentives were assigned to that ERT. Other ERT members were divided among the three ERTs to achieve a balance of representation from the research and programme communities and government agencies as well as of expertise in financial incentives and maternal health.

### Evidence acquisition

Two strategies were employed to acquire evidence for the Evidence Summit process: (i) a formal literature search conducted by public health professionals, and (ii) a call for evidence issued to members of the ERTs. Although it was recognized that high-quality evidence existed in both published and unpublished literature, the Core Group opted to limit the formal literature search to articles published in peer-reviewed scholarly journals, anticipating that relevant documents from the grey literature would be submitted by experts in the field in the subsequent call for evidence. The Core Group also decided to exclude documents based on research carried out in high-income countries. This decision reflected the thinking that research in high-income countries may have limited relevance to LMICs; if relevant research from high-income countries was identified by experts in the ERTs, it could be submitted during the call for evidence process.

**Figure 3.**
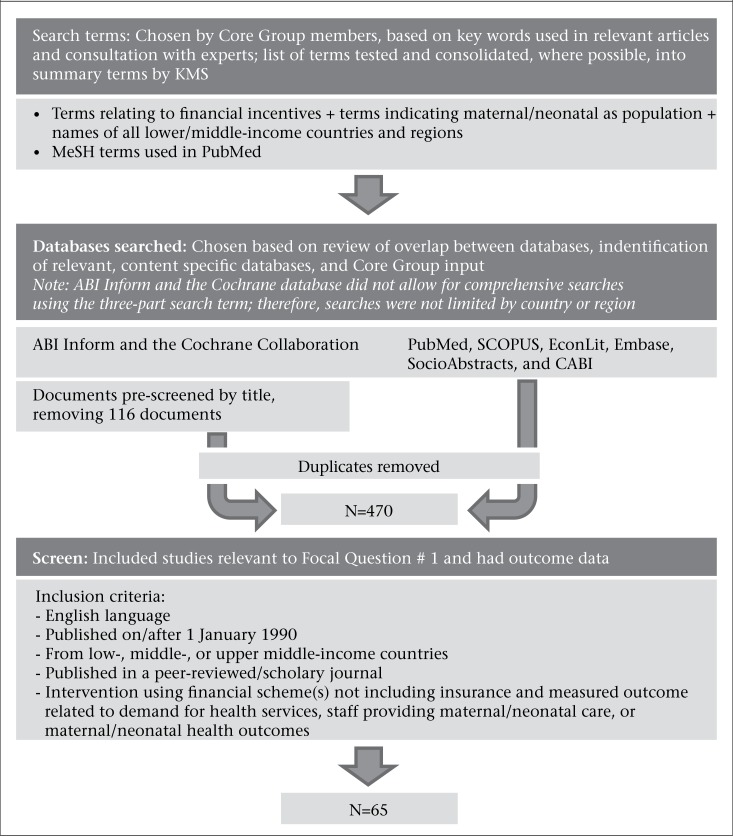
Initial search and screening process to obtain a core bibliography for the Evidence Summit

Figure 3 shows the results of the initial literature search. Knowledge Management Services (KMS) conducted the literature search, compiled the database, and conducted the screening and initial review of literature under contract arrangements with USAID in collaboration with the Core Group. The Core Group worked with KMS to select the search terms from key words identified in relevant articles and consultation with experts in the fields of maternal health and performance-based financing. The search strategy for the peer-reviewed literature combined terms for financial incentives (e.g. pay for performance, results based financing, performance based financing, performance based scheme*, results based incentive*, performance based contracting, results based contracting, paying for results, contracting in, contracting out, performance based aid, performance based disbursement, output based aid, output based financing, fee for service, cash transfer*, cash incentive*, financial incentive*, incentive*, incentive scheme*, token economy, reinforcement, voucher*, money to transport, transport fee*, subsidy, subsidies, subsidized care (subsidized-care), exemption*, waiver*, user fee*, user charge*, out-of-pocket payment*, coupon*, free care) with terms for maternal and neonatal health (e.g. matern*, antenatal, prenatal, preconception, intrapartum, perinatal, postpartum, postnatal, pregnan*, childbirth, child birth, birth, neonate, newborn, neonatal), and the names of countries and regions categorized as low or middle income by the World Bank.

The databases searched included ABI Inform, Cochrane Library, PubMed, SCOPUS, EconLit, Embase, SocioAbstracts, and CABI (Figure 3). Where possible, the following exclusion criteria were applied to the database searches:

- Documents not published in English
- Documents published before 1 January 1990
- Research carried out in high-income countries
- Magazine or newspaper articles
- Letters-to-the-Editor
- Obituaries
- Commentaries/recommendations not based on thorough literature reviews
- Book reviews
- Job postings
- Historical accounts

Following the pre-screen and removal of duplicates, the initial search of the peer-reviewed literature yielded a total of 470 papers. KMS then undertook a manual screening of the papers with two goals: (i) to exclude documents according to the basic exclusion criteria shown before, in case any such document had not been previously eliminated via the search limits and pre-screen, and (ii) to include only those papers with interventions directly related to the Focal Questions and that contained outcome data (Figure 3). The screening algorithm is provided in the USAID Evidence Summit web site http://www.usaid.gov/node/7186. The latter goal was achieved by including papers that described research on an intervention involving the use of one or more financial incentive(s) and included measurement of at least one outcome related to the demand for or utilization of maternal/neonatal health services by women, the performance of health professionals or organizations providing maternal/neonatal health services, or health-related maternal or neonatal outcomes that resulted from changes in the behaviour of patients or providers. Maternal/neonatal health services were defined as routine antenatal visits, special programmes for pregnant women (e.g. nutritional support, bednet provision, etc.), care for pregnant women suffering from an illness, intrapartum care, and other neonatal services through the first 28 days of life. Abortion and family planning services were excluded. The screening process was completed through a review of abstracts and resulted in the retention of 65 papers. In addition to excluding documents that did not meet the screening criteria, this screening step was also used in sorting documents into the type(s) of financial incentive(s) that were studied (supply-side, conditional cash transfers, or other demand-side mechanisms) and into the type(s) of outcome(s) measures that were studied (patients’ behaviour, service provision, or health outcome), effectively sorting documents into the matrix shown in Figure 2. This was done to assist the ERTs to subdivide their literature review tasks among their members.

**Figure 4.**
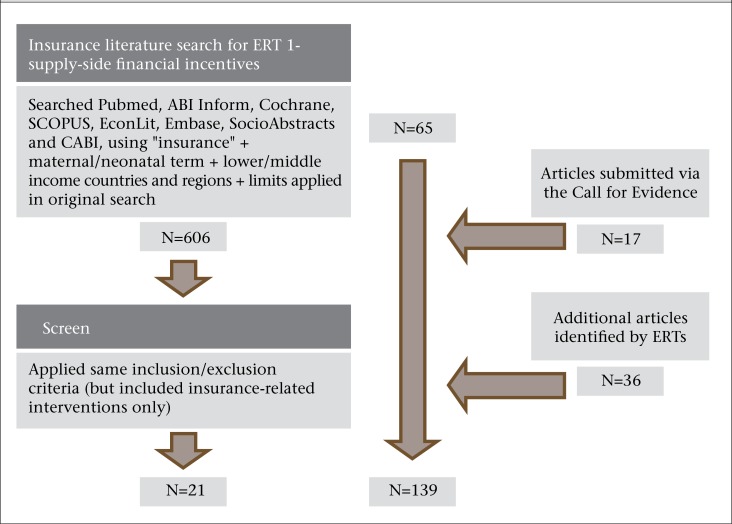
Diagram showing the steps for adding documents to the initial bibliography that include material on the role of insurance and the documents that were added through the Call For Evidence process

During the course of the evidence reviews, the ERT that was asked to examine the literature on supply-side incentives decided to include interventions that utilized insurance, necessitating a renewed literature search for documents on this topic that otherwise met the above criteria for inclusion (Figure 4). The initial search using insurance-related terms as shown in the figure yielded 606 papers but, after screening, the number of insurance documents was reduced to 21. Due to the difficulties in undertaking an insurance intervention study that includes a control group, articles reporting secondary population-level data that collected pre- and post-insurance policy implementation were also included.

To supplement the papers identified through the literature search, members of the ERTs were invited to submit documents they felt would help address the focal questions. This took place through a formal call for evidence prior to the Evidence Summit. It utilized an online document submission process in which ERT members were asked a series of questions about the documents they were submitting. The Call for Evidence submission protocol can be found on the Evidence Summit website: http://www.usaid.gov/node/7186. In selecting papers for submission, the ERT members were encouraged to review the exclusion criteria that had been applied to the initial literature search but they were not required to adhere to those criteria to enable the experts to include papers they deemed highly relevant to the focal questions. They were also advised that purely descriptive papers had not proven useful in previous USAID-supported Evidence Summits and that priority should be given to the following types of papers:

- Papers with primary data of high scientific quality with maternal or neonatal health outcomes, maternal healthcare-seeking behavioural outcomes, or provider behavioural outcomes that result from the application of a financial incentive.
- Papers summarizing interventions or evaluations
- Documents of relevance to low- and middle-income countries, even though these may describe work done in high-income countries
- Documents that had undergone peer review
- Systematic reviews
- Grey literature in the form of studies, reviews, or evaluations

A total of 25 documents were formally submitted through the call for evidence. Eight duplicates were removed, resulting in 17 additional documents (Figure 4). During the preparation of the evidence synthesis papers, ERT members were encouraged to cite other documents that they felt were relevant without submitting these through the form Call for Evidence Process. This resulted in an additional 36 documents. The final bibliography contained 139 documents. The geographic location where the studies were conducted for the final bibliography was as follows: Latin American Countries (n=28), Africa (n=48), Middle East and Southeast Asia (n=35), reviews or studies involving many LMICs (n=24), general papers about financial incentive-use in LMICs (n=4)

### Quality review process

After the screening and sorting processes were completed, ERT members were asked to assess the quality of the 65 papers derived from literature search and screening process and the 17 papers obtained through the Call for Evidence. A complex quality review framework for both empirical studies and programme evaluation documents had been developed previously for the Evidence Summit on Protecting Children Outside of Family Care (5). Experience from that summit suggested that a simpler quality review tool would be more practical for the purposes of this process, and so the question set was revised to a set of eight questions (visit http://www.usaid.gov/node/7186 for the text of the quality review questionnaire). ERT members were assigned sets of papers to review and asked eight questions concerning the types of equivalence of the comparator group, if any; the adequacy of the study design; the fidelity of the intervention; the validity and relevance of the outcome measures; the data analysis; the generalizability of the results; and evidence for the sustainability of the intervention. Each document was assigned to two ERT members for review. Of the 82 papers, 70 underwent a quality review with 28 papers reviewed by one reviewer, 33 papers reviewed by two reviewers, and 9 papers reviewed by three reviewers. Summary quality scores between 0 and 1 (0 for lower quality, 1 for higher quality) were derived for use by the ERT members in the later evaluation of evidence. The summary score did not penalize documents if reviewers skipped a question.

### Pre-summit meeting

The pre-summit was a technical working meeting held on 14 March 2012 in Bethesda, Maryland, hosted by the Eunice Kennedy Shriver National Institute of Child Health and Human Development. Members of the three ERTs came together to learn about the purpose of the Evidence Summit and its anticipated outcomes, discuss the initial review of the literature, develop work plans for producing an evidence synthesis and recommendations, and discuss strategies for increasing the impact of the Evidence Summit. The meeting involved the presentation of background material and a series of interactive roundtable discussions, and breakout sessions to facilitate the attainment of the pre-summit objectives.

Evidence packets summarizing the results of the quality review process were provided to ERT members at the pre-summit meeting. The packets were divided according to the ERT categories such that there was one packet for those papers relevant to supply-side incentives, one for those relevant to conditional cash transfers and one for those relevant to other demand-side incentives. Included in the evidence packets were the citations, abstracts, and individual responses and summary quality scores for each paper. During the pre-summit meeting, the ERTs used these reports to begin considering how well the identified literature addressed the two focal questions.

The Core Group outlined the expectations for the ERTs and for the products to be completed for the Evidence Summit in April, which included an evidence synthesis paper and a presentation summarizing the evidence synthesis, along with recommendations for policy, practice, and research. ERTs were encouraged to continue adding relevant documents to the bibliography throughout the development of the evidence synthesis papers. They were also provided with a framework for evaluating the evidence and making recommendations based on both evidence and expert opinion. This framework is described below. During the pre-summit period, the participants decided that insurance must be included as an FI mechanism, and an additional literature search followed to identify relevant studies.

### Evaluating evidence and making recommendations

ERTs were provided an evidence framework to assist them in drawing conclusions and making recommendations. The framework was developed for the Evidence Summit on Protecting Children Outside of Family Care and is described in more detail in a paper describing the methodology for that Evidence Summit (5). Evidence standards initially evolved from the medical field where physician's decision-making is determined primarily by data derived from randomized clinical trials which prove ‘efficacy’ for the individual patient. Evidence requirements for global health decisions and the evidence summits are more complex than those to support best practices guidelines for physician's/patient's decision-making. For global programming, the evidence must not only address ‘efficacy’ at the individual level or within a specific context but also ‘effectiveness’ at the community and population levels in differing locations and contextually-varied environments. Further, ‘sustainability’ at the country level is critical for country ownership and long-term feasibility. For governments of LMICs and donors, evidence on the feasibility of an intervention's implementation on a population basis and its cost-effectiveness are critical to investment and resource allocation decisions leading to the sustainability of the intervention. These three streams of evidence typically result from different research approaches; thus, varying methodologies are needed to evaluate the evidence generated by each stream. Therefore, the evidence evaluation approach for this Summit allowed for mixed research methodologies to incorporate relevant evidence targeted to the three crucial data streams of efficacy, effectiveness, and sustainability.

Given the complexity of global health and development questions, using both evidence-base as well as expert opinion is important in developing recommendations for policies and programmes that can maximize their impact. For some practices which have been widely and successfully implemented, there may not be rigorous controlled trials demonstrating their efficacy, much less their effectiveness and sustainability. It was also acknowledged that the quality of the research support for some interventions may not be very high, which is why ERT members were asked to rate the quality of the studies they were reviewing. In making recommendations for interventions to enhance the use and availability of maternal health services, ERT members were advised to use both evidence and expert opinion. They were also requested to consider the quality of the evidence and to make clear which recommendations relied more on expert opinion informed by field experience.

## EVIDENCE SUMMIT

ERTs were asked to prepare two presentations for the Evidence Summit: (i) a narrative evidence synthesis which contained a review of the evidence on the focal questions that pertained to the financial mechanism they were assigned to consider and (ii) a set of recommendations. The Evidence Summit was held in Washington, DC on 24-25 April 2012, and the agenda and list of participants are available on the Summit website (http://www.usaid.gov/node/7186). The agenda included panel discussions on important issues that were identified in multiple ERTs, such as unintended consequences of financial incentives, contextual issues, and a discussion on ethical issues involved in FI studies. Participants at the Evidence Summit included members of all of the ERTs as well as many additional experts in both science and practice of enhancing the provision and use of maternal health services and in using financial incentives. Notably, an effort was made to expand the involvement of LMIC representatives as participants in the Evidence Summit. The LMIC experts were included in roundtable discussions and included representatives, including His Excellency Mam Bunheng, Minister of Health, Cambodia; Dr. Fidele Ngabo, Director of the Maternal and Child Health Unit, Ministry of Health, Rwanda; Francis-Xavier Andoh-Adjei, Deputy Director of Strategy and Corporate Affairs, National Health Insurance Authority, Ghana; and Nazrin Oriakhail, Director, Malalai Maternity Hospital, Kabul, Afghanistan.

The Evidence Summit was designed to be a participatory process enabling the ERT to obtain feedback on their work and learn of issues or literature they may have missed. After each ERT provided an overview of their evidence synthesis and draft recommendations for policy, practice, and research, participants grouped by tables were invited to identify other documents that the ERTs should consider in their reviews and were asked to provide written individual and group feedback on coloured cards on recommendations, research priorities, and practices. Each table was asked to present the group feedback. Further, all the comment cards were collected, summarized, and provided back to the ERTs following the Evidence Summit.

After the Summit, the ERTs were asked to consider the comments and recommendations they received during the Summit and incorporate those into revised syntheses papers. In considering how to disseminate these findings, the leaders of the ERTs decided to produce five evidence papers organized by the financial incentives so that the evidence might be more easily utilized, i.e. by supply-side (performance-based incentives), insurance, conditional cash transfers, vouchers, and other demand-side interventions. These five papers form the core of the reports on the Evidence Summit that are published in this Supplement of the Journal.

### Conclusions

The commitment to evidence-informed practice, policies, and strategies to informing global health decisions is the first step in a long road to doing so, especially when demand for effective, efficient, global health services requires action now. Ideally, evidence-informed policy for global health necessitates the quality and standardized expert evaluation of the evidence for the critical challenges faced by governments of the low- and middle-income countries. These challenges, such as effective use of financial incentives to increase demand for and supply of quality maternal health services, are complex, multisectoral, and transministerial problems. Ideal literature to inform these problems would focus on the critical questions needed to inform government policies in the LMICs on sustainable, effective practices and strategies. The expertise in designing and conducting studies would parallel the multidisciplinary nature of the problem. The evidence would address relevant contextual issues so countries could know what works in the context of settings similar to theirs. Finally, studies on implementation science would follow standard guidelines for reporting similar to the consort guidelines.

The Evidence Summit process was developed to acquire and evaluate the available evidence to make recommendations for global health practice, policy, research prioritization, and strategy. Despite a thorough acquisition process, the identified literature for this Evidence Summit fell far short of ideal. Collectively, the literature did not address critical issues, such as sustainability, contextual issues, specificity around quality of care measures, or details on implementation that would enable reproducibility. Further, study designs usually fell short of needed information, such as causal attribution of the financial incentives to health outcomes, longitudinal neonatal and maternal health outcomes, and continued impact of FI; sustainability assessments, such as health value, cost-effectiveness, resource requirements, etc. A common challenge involved lack of coordinated multidisciplinary teams of economists and maternal health experts in planning and conducting studies. The majority of studies neglected to see the financial incentive in the paradigm of a dynamic system and, therefore, did not measure the impact on both supply and demand side of interventions. Few studies assessed unintended consequences resulting from financial incentive schemes. Further, it is quite clear that the research agenda must be informed, in part, by the policy-makers so that the research community can address the research around the key issues faced by policy-makers. Global health issues would benefit from a standardized evidence evaluation framework to guide the development of evidence and its evaluation and reporting. As part of the evidence to action strategy, USAID developed a concept for a consortium to bring economists and maternal health experts together to address the research needs in the area of financial incentives and maternal health. The idea for the formation of consortium was discussed with some of the organizations participating in the Evidence Summit including but not limited to USAID, NORAD, World Bank, Bill & Melinda Gates Foundation, USAID, and NICHD. Most opinions of those present were that an additional consortium was not needed to address the research needs because existing maternal health consortia could do so. These concerns are addressed in another paper in this Supplement (6).

This Evidence Summit drew lessons from the previous US Government Evidence Summit on Protecting Children Outside of Family Care (2), e.g. it did not attempt to use a search strategy to identify relevant grey literature which proved futile and instead used a “Call for Evidence” specifically asking for relevant grey literature. Clearly, the field would benefit from all rigorous studies being published in the peer-reviewed literature where it can be identified and used. Therefore, editors should be seeking and accepting such papers. Another adaptation was the lengthy quality review conducted on papers. During the previous Evidence Summit, the lengthy quality review deemed not useful to experts, and it was, therefore, replaced by a simple dichotomous assessment of quality. Many ERT members voiced concerns that the quality assessment was oversimplified. Further, although the ERT chairs reviewed and agreed to use a standard framework for rating quality and strength of recommendations, they found it quite challenging to do so.

Lack of ideal or robust evidence or an ideal framework in which to evaluate evidence should not deter the global health development and research communities from reviewing evidence around critical global health challenges. Doing so creates a transparent understanding of what is known enabling better decision-making processes, more targeted relevant research agenda while leveraging the tremendous resident expertise in the development, academic and multilateral communities.

## ACKNOWLEDGEMENTS

This work was carried out by the United States Federal Government Employees and persons supported through contractual mechanisms by the federal government. This is an open-access article under the terms of the Creative Attribution License, which permits unrestricted use, distribution, and reproduction in any medium, provided that the original authors and source are credited. Among the authors, ESH is supported by the National Institute of Allergy and Infectious Diseases and was on assignment with the Bureau for Global Health at USAID during the planning and execution of the Evidence Summit. ES is paid by Knowledge Management Services in Washington, DC. This organization received contractual funding from USAID for their work on the Evidence Summit. RR is currently paid by USAID. During the planning of the Evidence Summit, she was paid by the National Institutes of Health and was on assignment with the Bureau for Global Health at USAID. RLB is paid by Virginia Commonwealth University in Richmond, VA, USA. During the planning of the Evidence Summit, he was a Jefferson Science Fellow assigned to USAID and received supplemental support from the National Academy of Sciences. He currently receives part of his salary through an Intergovernmental Personnel Act arrangement with USAID. Under the direction of USAID staff, Knowledge Management Services designed the search and screening process, and USAID facilitated the publication of the manuscripts resulting from the Evidence Summit process. No funding bodies had any role in the data analysis or conclusions drawn from the literature reviews. The views and opinions expressed in this paper are those of the authors and not necessarily of the National Institutes of Health, the Department of Health and Human Services, or the Government of the United States.
